# Observation of number-density-dependent growth of plasmonic nanobubbles

**DOI:** 10.1038/srep28667

**Published:** 2016-06-29

**Authors:** Takashi Nakajima, Xiaolong Wang, Souvik Chatterjee, Tetsuo Sakka

**Affiliations:** 1 Institute of Advanced Energy, Kyoto University, Uji, Kyoto 611-0011, Japan; 2 Department of Energy and Hydrocarbon Chemistry, Kyoto University, Kyoto 615-8510, Japan

## Abstract

Interaction dynamics of laser pulses and nanoparticles are of great interest in recent years. In many cases, laser-nanoparticle interactions result in the formation of plasmonic nanobubbles, and the dynamics of nanoparticles and nanobubbles are inseparable. So far, very little attention has been paid to the number density. Here we report the first observation of number-density-dependent growth of plasmonic nanobubbles. Our results show that the nanobubbles growth depends (does not depend) on the number density at high (low) laser fluence, although the inter-particle distance in the solution is as long as 14–30 *μ*m. This cannot be explained by the existing physical picture, and we propose a new model which takes into account the pressure waves arising from nanoparticles. The numerical results based on this model agree well with the experimental results. Our findings imply that the number density can be a new doorknob to control laser-nanobubble as well as laser-nanoparticle interactions.

In the past decades intensive studies on the optical properties of nanoparticles (NPs) have led to various interesting applications in biophysics^1^,^2^ and many other fields^3^,^4^ as nicely reviewed in a recent article^5^. Most of these applications are based on the collective oscillations of unbound conduction electrons, known as surface plasmons. The energy acquired by the unbound electron through the formation of surface plasmon is transfered to the other electrons in tens of femtoseconds, and then to the lattice within a few picoseconds, which leads to the heating of NPs. The heat diffusion originating from NPs goes outwards as well as inwards, as a result of which the surrounding medium is also heated^6^,^7^,^8^,^9^,^10^,^11^,^12^,^13^. In many applications the surrounding medium is water (or liquid), and as a natural consequence of the plasmonic heating of NPs, plasmonic nanobubbles (NBs) are formed^14^,^15^,^16^,^17^,^18^. Plasmonic NBs are very transient, and exhibit very different behaviors compared with the surface NBs^19^,^20^ which are found to be very stable. The dynamics of NPs and NBs upon laser irradiation onto the NPs depends not only on the incident laser fluence^8^ but also the laser wavelength^21^. Similarly, the laser wavelength to induce optical breakdown of water without NPs has been found to be an important parameter to control the radius of NBs, which may lead to the improvement of cell surgery^22^.

Since the formation of plasmonic NBs is a consequence of laser-NP interactions in the solution, the dynamics of NPs and NBs are inseparable, and therefore, the fate of NPs upon laser irradiation is strongly related to the fate of NBs. For more efficient use of NPs and NBs, the NP solution with higher number density, or laser pulse with higher fluence, or both may be sometimes desired. In this aspect, we point out that very little attention has been paid, so far, to the choice for the number density of NPs in the solution.

In this work we report the first observation of number-density-dependent growth of plasmonic NBs upon irradiation of laser pulses. The experimental technique we develop here is capable of measuring the time-dependent dynamics of plasmonic NBs with nanosecond time-resolution, which could provide much more insight into the dynamics of NBs, and the technique could be easily extended for the femtosecond time-resolution only if a femtosecond laser is available. The number density of NPs we employ in this work is quite modest, and in the range of 10^7^~10^8^/mL. The dynamics of NBs are monitored through the transient change of extinction cross section of NBs, which is converted to the temporal change of NB radius with the aid of Mie theory. Our results clearly demonstrate that the growth of NBs irradiated by the high-fluence laser pulse is significantly suppressed if the initial number density of NPs is high (but still in the order of only ~10^8^/mL, corresponding to the inter-particle distance of ~14 *μ*m), while the NB growth under the low-fluence laser pulse hardly depends on the number density. This cannot be explained by the existing physical picture, and we propose a new model which takes into account the influence of the pressure waves arising from NPs. The numerical results based on this model agree well with the experimental results.

## Results

### Time evolution of nanobubble radius

**Figure 1:**
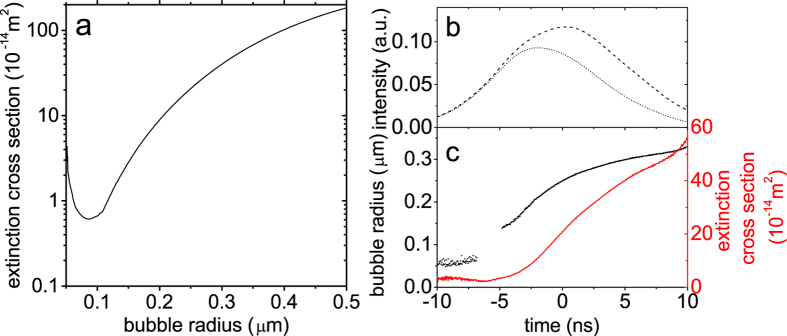
Procedure to obtain the time evolution of bubble radius from the raw data of temporal pulse profiles before and after the cuvette. (**a**) Extinction cross section of an air bubble at 532 nm as a function of bubble radius calculated by the Mie theory. A single Ag NP of 50 nm radius is assumed to be at the center of the bubble as a core. (**b**) Typical temporal profiles of 532 nm pulses before (dashed) and after (dotted) the cuvette. (**c**) Time evolution of extinction cross section (red) calculated from the curves in graph (**b**) and time evolution of bubble radius (black) obtained using the red curve and graph (**a**). Note that the gap at around −7 ns in graph (**c**) is caused by the nonmonotonicity of the change of extinction cross section seen in graph (**a**) as a dip for the small bubble radius.

The Ag NPs solution we employ for the experiments is a commercial product from nanoComposix, and has good size uniformity (99 ± 8.2 nm diameter) and low ellipticity (Supplementary Fig. S1) with 2 mM citrate to prevent aggregation. We dilute it with distilled water to obtain the AgNPs solutions with desired number densities. Upon irradiation of the 532 nm laser pulse onto the Ag NPs solution (Supplementary Fig. S2) NBs are formed. The produced NBs has a spherical shape (Supplementary Fig. S3), and essentially contains water vapor (refractive index ~1) with a Ag NP as a core. Of course the Ag NPs may be melted and even vaporized in the NBs during the irradiation of 532 nm laser pulses. However, the closest transition wavelength of Ag atoms is 328 nm, which is very much away from the wavelength of the laser pulse, and therefore the Ag vapor may be considered to be transparent to the 532 nm laser pulses. Note that the formation of NBs upon irradiation of a laser pulse is a transient process, and using a simple but new setup with a single laser pulse (Supplementary Fig. S2), the time-dependent change of NBs radii can be detected through the change of the temporal profile of the 532 nm pulse before and after the cuvette. This procedure is illustrated in Fig. 1a–c. Figure 1a shows the extinction cross sections of water vapor NBs at 532 nm calculated with the Mie theory (ScatLab software) as a function of bubble radius. Using the raw experimental data of temporal pulse profiles before and after the cuvette shown in Fig. 1b, we obtain the time-dependent extinction cross sections as shown by the red curve in Fig. 1c, which is finally converted to the time-dependent bubble radius shown by the black curve in the same panel with the aid of Fig. 1a. Note that the gap appears at around −7 ns in the temporal change of bubble radius shown in Fig. 1c, which is due to the fact that the extinction cross section cannot be uniquely mapped onto the bubble radius when the bubble radius is very small, as we see in Fig. 1a at ~0.1 *μ*m. From Fig. 1c we can clearly see that the NB radius monotonically increases during the irradiation of the 532 nm pulse.

### Nanobubble growths at different number densities

**Figure 2:**
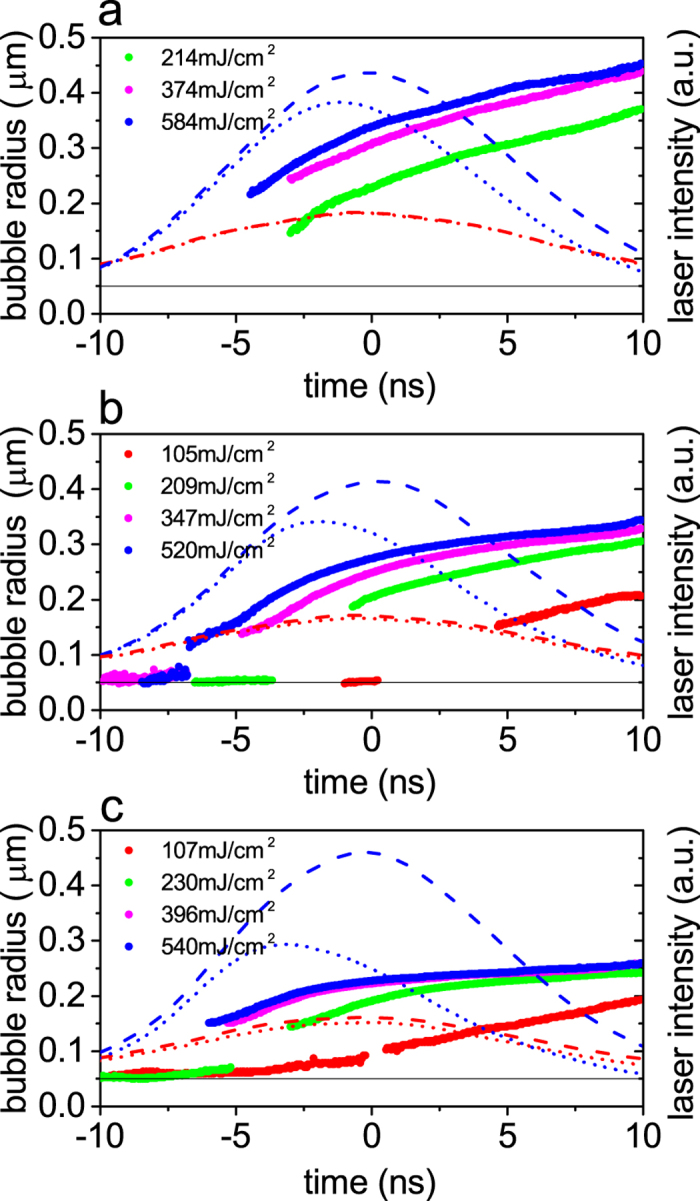
Time evolution of bubble radius for different laser fluences and number densities of Ag NPs. The dashed and dotted curves in each panel are the temporal profiles of 532 nm pulses before and after the cuvette, and the different colors indicate the different laser fluences. The number densities of Ag NPs are (**a**) 3.7 × 10^7^/mL, (**b**) 1.2 × 10^8^/mL, and (**c**) 3.7 × 10^8^/mL, respectively.

Now, we vary the laser fluences and number densities of Ag NPs in the solution and repeat the measurements, after which the data analysis is performed by the procedures described in Fig. 1a–c. The results are summarized in Fig. 2. What we can see from these results is that, for any number density of Ag NPs in water, for example in Fig. 2a, the NBs start to grow at earlier time and the final bubble radius is larger for higher laser fluence. This is simply because more heating of surrounding water as well as Ag NPs takes place when the incident laser fluence is higher^11^. This is already well-known. However, in case of high laser fluence, we find that the NBs growth has a striking dependence on the number density of Ag NPs. For instance, compare the NB radius at 10 ns in Fig. 2a,c at 584 mJ/cm^2^ and 540 mJ/cm^2^, respectively. The NB radius is 0.45 *μ*m for the former and 0.26 *μ*m for the latter. In contrast, the NB radius is very similar, irrespective of the number density, when the laser fluence is sufficiently low. For instance, compare the results in Fig 2b,c at 105 mJ/cm^2^ and 107 mJ/cm^2^, respectively. At 10 ns the NB radius is ~0.2 *μ*m for both cases. We note that the similar measurement was not possible for the number density of 3.7 × 10^7^/mL, because the difference of the temporal profile of the 532 nm pulse before and after the cuvette (red dashed and dotted lines in Fig. 2a) was too small to reliably determine the time evolution of bubble radius.

### Nanobubble radius at the end of the pulse

**Figure 3:**
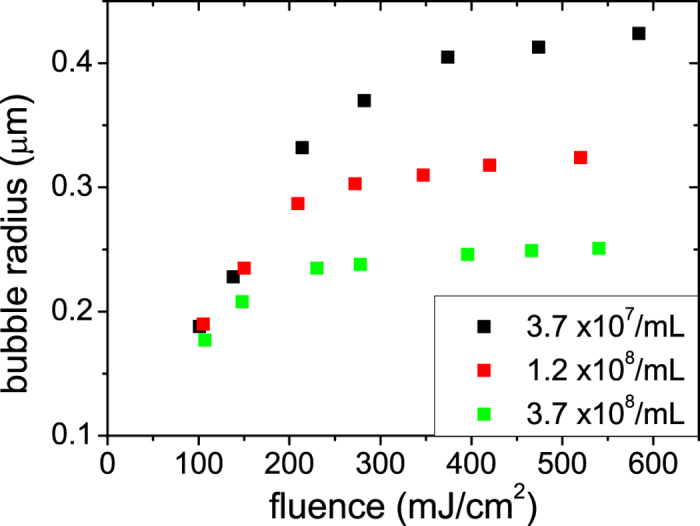
Bubble radius at 7 ns as a function of laser fluence for different number densities of Ag NPs.

**Figure 4:**
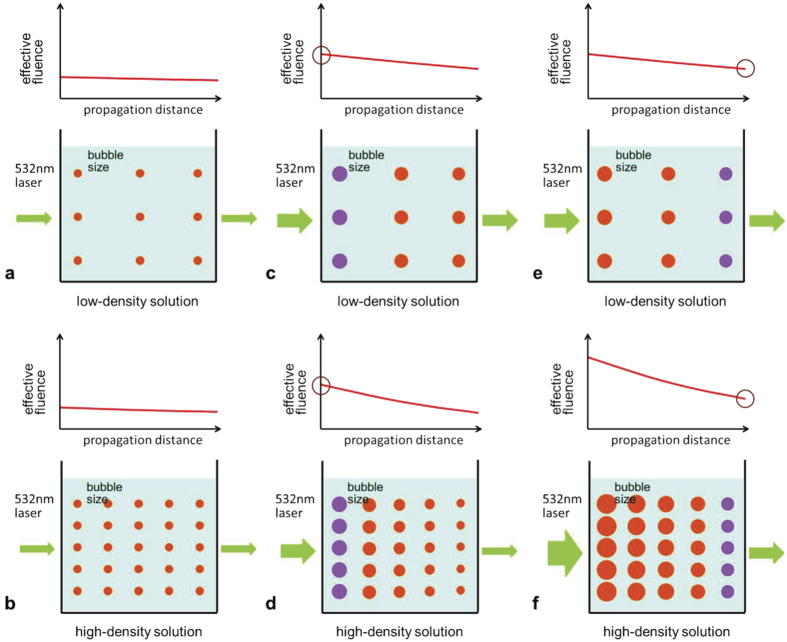
Illustration of NBs at the end of the laser pulse to explain why the simple consideration of effective laser fluence of 532 nm laser pulse in the AgNPs solutions *cannot* explain the number-density-dependent growth of NBs presented in Figs 2 and 3. (**a**,**b**) illustrate the NBs at low laser fluence for the low and high density solutions, respectively, while (**c**,**d**) show the similar at high laser fluence. In each (**a**,**b**) and (**c**,**d**), the laser fluence is chosen to be the same at the *entrance* of the cuvette. (**e**,**f**) illustrate the NBs at high laser fluence for the low and high density solutions, respectively, with laser fluence chosen to be the same at the *exit* of the cuvette.

To highlight the number-density-dependent growth of NBs we replot the bubble radius at a chosen time, 7 ns, as a function of laser fluence for different number densities by referring to Fig. 2a–c, and the results are shown in Fig. 3. When the laser fluence is low (~100 mJ/cm^2^) the bubble radius is about 0.18 *μ*m, and it does not depend on the number density. As the laser fluence increases, the bubble radius is becoming different for different number densities, and if we compare the bubble radius under the same *incident* laser fluence, it is smaller for the higher number density than that for the lower number density. For instance, at the laser fluence of ~550 mJ/cm^2^, the bubble radii are, respectively, 0.41, 0.32, and 0.25 *μ*m, for the number densities of 3.7 × 10^7^, 1.2 × 10^8^, and 3.7 × 10^8^/mL. Note, however, that these comparisons do not bring any surprise, because we can easily explain the above findings in Fig. 3, at least qualitatively, by the well-known physical picture, as illustrated in Fig. 4a–d. Namely, if the incident laser fluence is low, the laser pulse is hardly attenuated, and accordingly the the size of NBs is practically the same everywhere for both low and high density solutions, as illustrated in Fig. 4a,b. This explains why the bubble radius in Fig. 3 does not depend on the number density at the low laser fluence. If the incident laser fluence is high (Fig. 4c,d), the radii of NBs near the entrance of the cuvettes are supposed to be the same for both low and high density solutions (filled violet circles in Fig. 4c,d). Near the exit of the cuvette, however, the radii of NBs are smaller for the high density solution than those for the low density solution, because the laser pulse in the high density solution is significantly attenuated during propagation. This explains why, in Fig. 3, the bubble radius for the 3.7 × 10^8^/mL solution is smaller than that for the 3.7 × 10^7^/mL solution if the incident laser fluence is high. Thus, it appears that all we see in Fig. 3 can be explained by the existing physical picture, as illustrated in Fig. 4a–d.

However, a closer examination of Fig. 3 reveals the very unusual NBs growth at different number densities: We now compare the bubble radius under the same laser fluence at the *exit* of the cuvette. For instance, the incident laser fluence of 214 mJ/cm^2^ for the 3.7 × 10^7^/mL solution and that of 540 mJ/cm^2^ for the 3.7 × 10^8^/mL solution result in the similar laser fluence of 200~240 mJ/cm^2^ at the exit of the cuvette, since the transmittance of the 532 nm pulse after the cuvette is close to 100% for the former and ~45% for the latter solutions. A naive guess based on the existing physical picture, as illustrated in Fig. 4e,f, suggests that the ensemble-averaged bubble radius for the former solution (Fig. 4e) must be smaller than that for the latter solution (Fig. 4f). But this turns out to be wrong, as we see in Fig. 3. The bubble radii at 7 ns are 0.34 and 0.25 *μ*m, respectively, for the 3.7 × 10^7^ and 3.7 × 10^8^/mL solutions. Clearly, a simple consideration of the effective laser fluence based on the existing physical picture (Fig. 4e,f) cannot explain why the NBs growth for the 3.7 × 10^8^/mL solution at the incident laser fluence of 540 mJ/cm^2^ is strongly suppressed compared with that for the 3.7 × 10^7^/mL solution at the incident laser fluence of 214 mJ/cm^2^.

### Pressure waves

**Figure 5:**
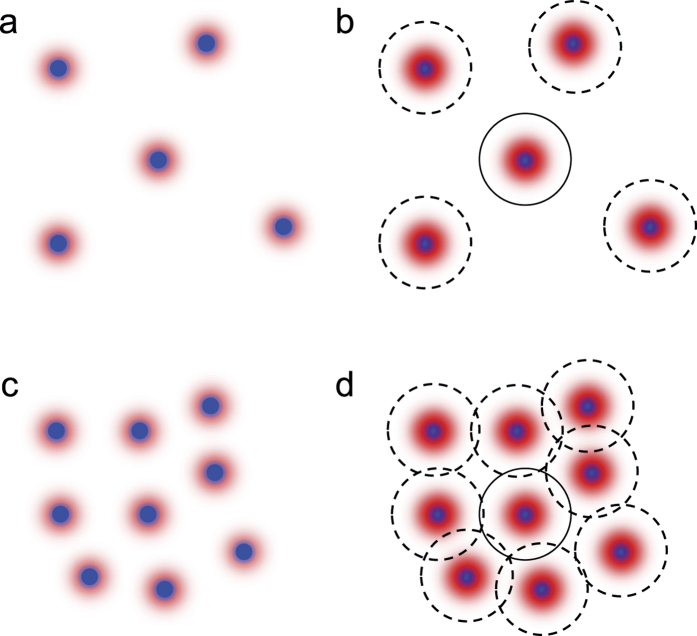
Role of the pressure waves during the NBs growth for the cases in which the incident laser fluence and the number density of NPs are, respectively, (**a**) low and low, (**b**) high and low, (**c**) low and high, and (**d**) high and high. In (**b**,**d**) the dashed circles stand for the wavefronts of pressure waves at several ns. Note that in (**d**) the pressure waves originating from the NPs in the neighborhood can influence the growth of the other NBs even in the short time range (several ns) as studied in this work.

We attribute this unexpected behavior of the NB growth at different number densities to the effect of the pressure waves^23^,^24^,^25^,^26^. When the NPs are suddenly heated by laser pulses, NBs are formed around NPs. Since the formation of NBs is so sudden the pressure waves are produced. In the existing theory to describe the time evolution of the bubble it is usually assumed that the bubble is isolated. This is a reasonable assumption for the laser ablation of a solid target in a gas^23^ and liquids^24^,^25^,^26^,^27^. In our case it also appears, at first glance, that the bubbles are well-isolated if we recall that the inter-particle distance at the number densities studied in this work is 14–30 *μ*m (second column of Table 1), which is far larger than the bubble radius and there should be no change in the optical property of NPs at this inter-particle distance^28^. There is a possibility, however, that the pressure wave produced around a certain NP reaches the adjacent NBs during the irradiation of the laser pulse. The mean travel time of the pressure wave to the adjacent NPs is also written in the third column of Table 1. We point out that the speed of the pressure wave in water is at least faster than the speed of sound in water, i.e., 1500 m/s, and it can be even 3000–4500 m/s, as measured for the case of optical breakdown in water by the 6 ns pulse with 10 mJ energy^29^, while the expansion speed of the bubble can be much slower and may be of the order of tens or hundreds of m/s^7^,^27^. It may be that the outward pressure wave originating from a certain NPs acts as the *inward pressure wave* to the adjacent NBs to suppress the growth of NBs. This hypothesis is qualitatively consistent with our observation (Fig. 3) that (1) when the incident laser fluence is low (~100 mJ/cm^2^) the bubble radius at the end of the laser pulse does not depend on the number density, while (2) when the incident laser fluence is high (~500 mJ/cm^2^) it becomes smaller for the NP solution at the higher number density. The above interpretation is illustrated in Fig. 5 for four representative cases.

To confirm our hypothesis we carry out the numerical calculations by solving the Rayleigh-Plesett (RP) equation^30^,^31^,^32^ with appropriate modifications to take into account the effects of the pressure waves from the surrounding NPs (Supplementary Note 1), and the parameters we must know in advance are the peak pressure, *p*_*s*0_, and initial bubble speed, . For our specific case, those parameters serve as fitting parameters to better reproduce the experimental results. Unlike in the case of laser ablation and laser-induced breakdown in a liquid by which a single bubble is produced, many NBs are produced in our case, and moreover, those NBs are born at different times from the different NPs during the nanosecond laser pulse. Accordingly we perform the ensemble average of the calculated quantities (Supplementary Note 2)

**Figure 6:**
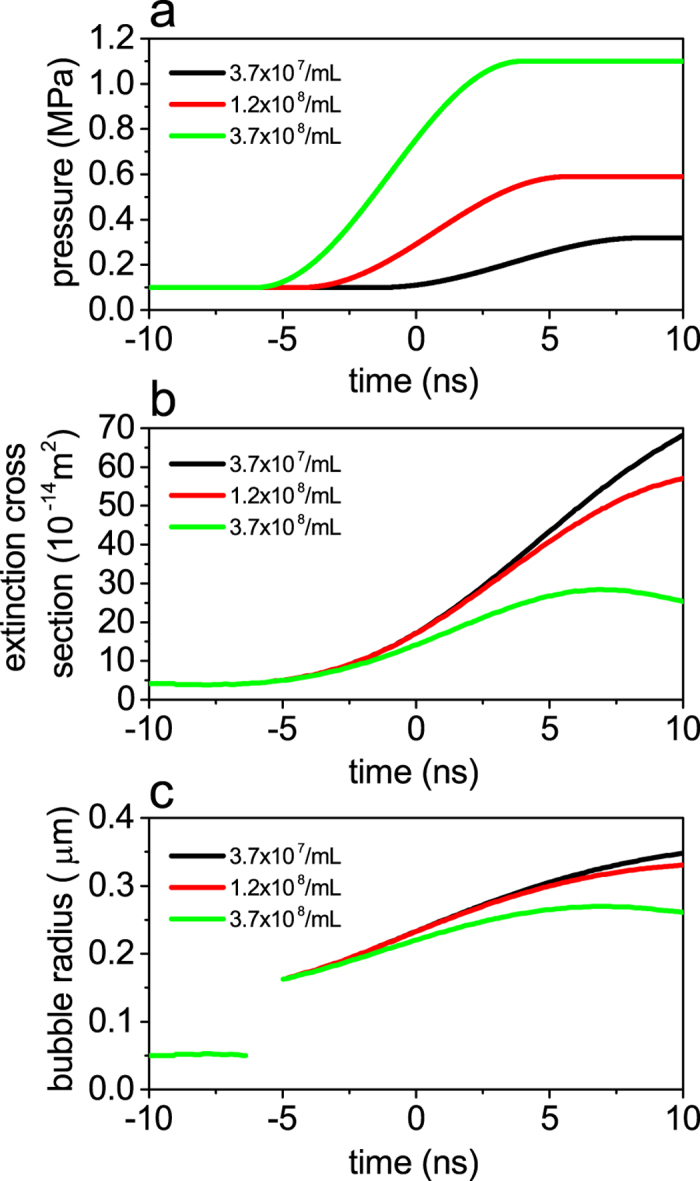
Calculated time evolution of NBs after the ensemble average for the case of the highest laser fluence, 520~580 mJ/cm^2^, by taking into account the influence of the pressure wave. (**a**) Temporal profiles of the pressure wave we assume for different number densities of Ag NPs. (**b**) Time evolution of extinction cross sections. (**c**) Time evolution of NB radius. In all figures the black, red, and green curves correspond to the number densities of 3.7 × 10^7^, 1.2 × 10^8^, and 3.7 × 10^8^/mL, respectively.

After we have tried several different values for the peak pressure *p*_*s*0_ and the initial bubble speed , we find that the bubble dynamics calculated with a smooth increase to the peak pressure wave of 1 MPa (Fig. 6a) and the initial bubble speed of 800 m/s can well reproduce the growth of NBs for the case of the highest number density of Ag NPs (3.7 × 10^8^/mL) under the highest laser fluence (~540 mJ/cm^2^). To ensure that those adjusted parameters are also appropriate to describe the bubble dynamics at the lowest (3.7 × 10^7^/mL) and medium (1.2 × 10^8^/mL) number densities under the similar laser fluence we solve the RP equation with the consistently scaled peak pressure (Supplementary Note 1) and the identical initial bubble speed, since the former (latter) parameter should (should not) depend on the number density. The numerical results of ensemble-averaged extinction cross section and bubble radius are shown in Fig. 6b,c. The trend we see in the calculated results reasonably agrees with the experimental results for the case of the highest laser fluence (~540 mJ/cm^2^) shown in Fig. 2, and this confirms that the physical origin of the number-density dependence of the NB growth is the pressure waves arising from the surrounding NPs.

## Discussion

We have studied the number-density-dependent growth of plasmonic NBs in the Ag NP solution. When the incident laser fluence is sufficiently high, the growth of NBs in the high number density Ag NP solution is suppressed compared with that in the low number density Ag NP solution. In contrast, the growth of NBs does not depend on the number density of NPs when the incident laser fluence is low. A simple argument based on the existing physical picture (Fig. 4e,f) cannot explain the number-dependent-growth of plasmonic NBs. We attribute this unexpected behavior to the effects of the pressure waves produced by NPs upon the formation of NBs. The pressure waves can reach the adjacent NBs within a few nanoseconds, and impose inward pressure to the adjacent NBs so that the the growth of the NBs is suppressed (Fig. 5). Our numerical results support this interpretation. Clearly, the number-density-dependent growth of NBs we have observed during the first several nanoseconds upon laser irradiation would influence their fate until they collapse, whether the employed pulse is long or short, and perhaps more importantly, whether the employed NPs are silver or gold or any other kind. Our findings can offer a new doorknob to control laser-NB as well as laser-NP interactions with a number density of NPs as an unexplored control parameters.

## Methods

### Extinction Spectrum of Ag NPs before the laser pulse

The Ag NP solution we employ is a commercial product from nanoComposix. It has good size uniformity (99.1 ± 8.2 nm diameter) and low ellipticity. The initial number density of Ag NPs is 3.7 × 10^9^/mL with 2 mM citrate to prevent aggregation. For our experiment we dilute the solution with distilled water in a well-controlled manner so that we can prepare the sample solution at the desired number density. To confirm that the number density of diluted NPs is correctly evaluated, we measure the extinction spectrum of 10× diluted Ag NP solution with a commercial CCD spectometer (Ocean Optics, USB2000+) and a white light source (Ocean Optics, USB-ISS-UV-VIS) (Supplementary Fig. S1). For comparison we also calculate the extinction spectrum at this number density with the Mie theory (Supplementary Fig. S1). Although the measured spectrum is a bit different from the calculated one in the region close to resonance, the overall shapes are similar, in particular around 532 nm. The overall agreement between the measured and calculated extinction spectra confirms that the number densities of the samples we prepare are correctly evaluated.

### Measurement of the time-dependent extinction

To measure the time-dependent extinction of Ag NPs dispersed in water, we detect the temporal profiles of the laser pulse before and after the Ag NPs solution (Supplementary Fig. S2). The second harmonic (532 nm) of the Q-switched Nd:YAG laser (Spectra-Physics, Quanta-Ray, 10 ns) is introduced into the 50 *μ*L quartz cuvette (1 cm length) filled with the Ag NP solutions at a few different number densities as described above. The timing of 532 nm laser pulses is synchronized with other instruments by the home-made electronic controller. In order to realize the uniform irradiation throughout the entire cuvette we use the well-collimated 532 nm beam without focus after reducing its diameter from 8 mm to 0.6 mm with a diaphragm placed before the cuvette. The transverse profile of the resulting beam is Gaussian (inset of Supplementary Fig. S2). Using the wedged beam samplers we measure the temporal profiles of 532 nm pulses before and after the cuvette with two photodiodes (ThorLabs, DET10A). The data from the photodiodes are transferred to the digital oscilloscope (Tektronix, TDS2014), and finally stored in the computer. The main portion of the pulse energy after passing through the cuvette is recorded by an energy meter (ThorLabs, ES111C & PM100D), which is also stored in the computer for the data processing. Since the irradiation of single laser pulse can result in the size reduction of NPs^33^,^34^ we replace the NP solution after the irradiation of each laser pulse so that the fresh and hence a well-defined NP solution interacts with every single laser pulse. From the measured temporal profiles of 532 nm pulses before and after the cuvette we can find the temporal change of transmission induced by the irradiation of single 532 nm pulse to the Ag NPs solution. Obviously the precise adjustment of time zero for the signals of the two photodiodes is crucial for our measurement. This can be done by taking into account for the ~10 cm distance between the two beam samplers, which results in the ~0.3 ns delay between the measured pulse shapes before and after the cuvette. To compensate for this time lag, we have shifted the temporal profile of the 532 nm pulse after the cuvette by 0.3 ns to the forward direction.

### Data analysis for the time-dependent extinction cross sections

From the experimentally measured time-dependent extinctions the time-dependent extinction cross sections during the single laser pulse can be calculated in the following way: We first measure the temporal profiles of 532 nm pulses before and after the cuvette, *I*_1_(*t*) and *I*_2_(*t*), with the cuvette filled with distilled water, and calculate the transmission by *T*_*water*_(*t*) = *I*_2_(*t*)/*I*_1_(*t*). Then, we replace the distilled water with the Ag NP solution at a well-defined number density, and again measure *I*_1_(*t*) and *I*_2_(*t*) to calculate the transmission, *T*_*Ag*_(t). Using those quantities we can calculate the time-dependent extinction cross sections at 532 nm as


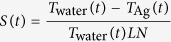


where *L* is the length of the cuvette and *N* is the number density of Ag NPs in water. Note that the above analysis is meaningful only if the NP solution before the laser irradiation is under the well-defined conditions in that the NPs have a known size and uniform distribution in water and the number density is also known. This is why our measurement is a single-shot basis with a fresh Ag NP solution for every 532 nm pulse.

To demonstrate the validity of our experimental scheme (Supplementary Fig. S2) and the data processing based on equation 1, we first perform the test measurements using the Ag NP solution at the number density of 3.7 × 10^8^/mL and the incident laser fluence of 180 mJ/cm^2^ with linear polarization. The result is shown by the black curve in Supplementary Fig. S3. Using *I*_1_(*t*) and *I*_2_(*t*) which are shown by the dashed and dotted curves, respectively, in Supplementary Fig. S3, we can calculate the time-dependent extinction cross sections with the aid of equation 1. The obtained curve for the extinction cross section is a bit noisy at time before −7 ns. This is due to the limited quality of the obtained signals of temporal pulse profiles. Similar is true at time after 7 ns. The dashed horizontal line in Supplementary Fig. S3 indicates the extinction cross section calculated by the Mie theory without taking into account the heating and formation of NBs, which corresponds to the weak excitation limit. In order to ensure that there is no deformation of NPs and NBs due to the specific choice of laser polarization, we also carry out similar measurements with elliptically and circularly polarized 532 nm pulses using a quarter-wave plate. The results are also shown by the red and blue curves in Supplementary Fig. S3. We can see that the time-dependent extinction cross sections by linearly, elliptically, and circularly polarized 532 nm pulses are practically the same in the time range between −7 and 7 ns. This clearly shows that we do not have to worry about the influence of employed laser polarization on the time-dependent extinction cross sections^35^. Accordingly we will only employ linearly polarized 532 nm pulses for the rest of the results shown in this work.

### Numerical calculations.

To simulate the temporal dynamics of the nanobubble we solve the Rayleigh-Plesset (RP) equation^27^,^30^,^31^,^32^ which is often employed to understand the dynamics of the single isolated bubble produced by laser-induced breakdown^22^,^29^, laser ablation of a solid target in a liquid^24^,^25^,^26^,^27^, and even cavitation dynamics of NBs formed by the irradiation of femtosecond pulses onto the gold NPs in water^7^. In our case, it is essential to take into account the influence of the pressure waves from the surrounding NPs, and the RP equation has to be appropriately modified, as we describe in Supplementary Note 1.

### Ensemble average of the numerical results

Since the employed laser pulse has a nanosecond duration different NBs are born from different NPs at different times during the laser pulse, and as a consequence, what we experimentally observe is the ensemble-averaged radius of many NBs. After these considerations, it is clear that we must perform the ensemble average of the numerical results (Supplementary Note 2) by recalling that it is not the bubble radii but the extinction cross sections that may be arithmetically averaged. That is, we numerically solve the RP equation many times to obtain the time-dependent bubble radii at a certain number density of NPs under the influence of the pressure wave as assumed in Fig. 6a for different onset times of the NBs, and cast the calculated bubble radii into the extinction cross sections using the Mie theory. The extinction cross sections are then ensemble averaged with weighting factors proportional to the number of NBs born at different times (Supplementary Note 2). The number of NBs born at different times is assumed to be proportional to the instantaneous laser intensity. The ensemble-averaged extinction cross section is finally recast into the bubble radius. Note that this kind of ensemble effect does not exist if the employed laser pulse into the NP solution has a much shorter (picosecond or femtosecond) duration^7^ or a single NB is produced by laser ablation onto a solid target placed in liquid^27^.

## Additional Information

**How to cite this article**: Nakajima, T. *et al*. Observation of number-density-dependent growth of plasmonic nanobubbles. *Sci. Rep*. **6**, 28667; doi: 10.1038/srep28667 (2016).

## Supplementary Material

Supplementary Information

## Figures and Tables

**Table 1:** Mean nanoparticle distance (defined as a Wigner-Seitz radius) and mean travel time of the pressure wave for different number densities of Ag NPs in water.

Number density (cm^−3^)	Mean nanoparticle distance (*μ*m)	Mean travel time (ns)
3.7 × 10^7^	30	8.6
1.2 × 10^8^	20	5.7
3.7 × 10^8^	14	4

The speed of the pressure wave in the Ag NP solution is assumed to be 3500 m/s^29^.
