# Supplementary material for: Observation of number-density-dependent growth of plasmonic nanobubbles
Source: Sci Rep. 2016 Jun 29;6:28667. doi: 10.1038/srep28667 (PMC4926106; doi:10.1038/srep28667)
Supplement: Supplementary Information [file srep28667-s1.pdf]

Supplemental material for "Observation of number-density-dependent growth of plasmonic nanobubbles "

Takashi Nakajima,[1,*] Xiaolong Wang,[1] Souvik Chatterjee,[1] and Tetsuo Sakka[2]

[1]*Institute of Advanced Energy, Kyoto University, Uji, Kyoto 611-0011, Japan*

[2]*Department of Energy and Hydrocarbon Chemistry, Kyoto University, Kyoto 615-8510, Japan*

*Electronic address: nakajima@iae.kyoto-u.ac.jp

Supplementary Figures

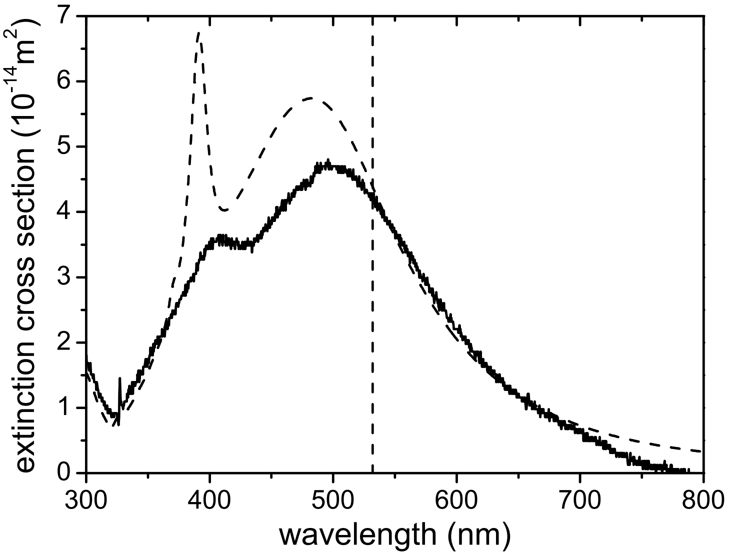

FIG. S1: Measured (solid) and calculated (dashed) extinction spectra of Ag NPs with ∼100 nm diameter dispersed in water. The dashed vertical line denotes the wavelength of the laser we use, which is close to the plasmon resonance (509 nm) of Ag NPs. The peak at 400 nm is due to the quadrupole resonance.

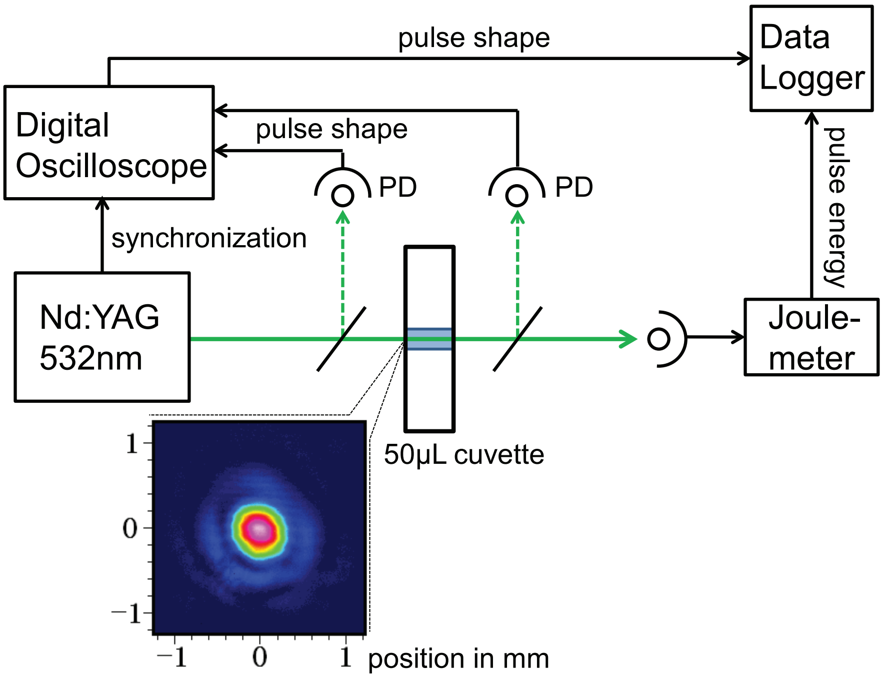

FIG. S2: Experimental setup for the measurement of time-dependent extinction cross sections of Ag NPs dispersed in water. For each laser shot we prepare a fresh NP solution, and measure the temporal profiles of single nanosecond 532 nm pulse by a pair of photodiodes before and after the cuvette. The inset shows the transverse intensity profile of the 532 nm pulse, and its borders denote the ∼2.2 mm side edge of the 50 μL cuvette.

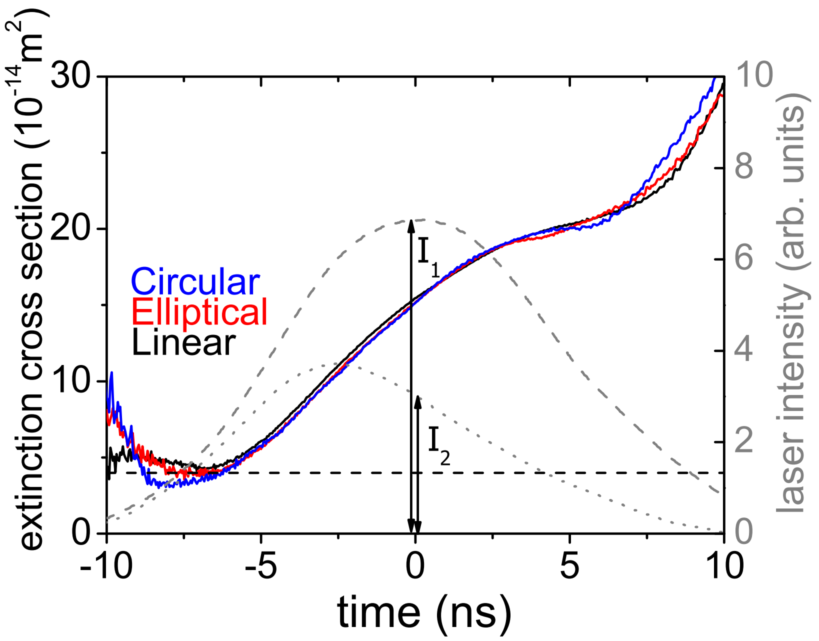

FIG. S3: Time evolution of the extinction cross section of Ag NPs in water irradiated by the single 532 nm pulse. The number density of Ag NPs is 3.7×10^8/mL and the laser fluence is 180 mJ/cm^2. Employed laser pulses are linearly (black), elliptically (red), and circularly (blue) polarized. The fact that the extinction cross sections by differently polarized laser pulses behave practically the same imply that the Ag NP and NBs have spherical shapes. The two grey curves illustrate the typical temporal profiles of 532 nm pulses before (dashed) and after (dotted) the cuvette filled with a Ag NP solution and provide the guidance of the temporal scale. The horizontal dashed straight line at $4.4{\times}10^{-14}$ m^2 indicates the extinction cross section calculated by the Mie theory, and therefore corresponds to the weak excitation limit.

Supplementary Note 1

Modified Rayleigh-Plesset equation

To test our hypothesis that the number-density-dependent NB growth originates from the pressure waves from the surrounding NPs, we carry out the calculations by solving the Rayleigh-Plesset (RP) equation [1–3] which describes the growth of a bubble in a liquid. Although the RP equation is often employed to understand the dynamics of the single isolated bubble produced by laser-induced breakdown [4, 5], laser ablation of a solid target in a liquid [6–8], and even cavitation dynamics of NBs formed by the irradiation of femtosecond pulses onto the gold NPs in water [9], this is a first attempt, to our knowledge, to apply the RP equation for the description of NBs by taking into account the influence of the pressure waves from the surrounding NPs.

Introducing the density, viscosity, and surface tension of the liquid, ρ_L, υ, and S, respectively, our modified RP equation to describe the temporal change of the bubble radius, $R(t)$, reads [1],

$$\frac{p_B(t) - p(t)}{\rho_L} = R(t)\frac{d^2R(t)}{dt^2} + \frac{3}{2}\left[\frac{dR(t)}{dt}\right]^2 + \frac{4\upsilon}{R(t)}\frac{dR(t)}{dt} + \frac{2S}{\rho_L R(t)} \tag{1}$$

where the pressure inside the bubble, $p_B(t)$, is assumed to be well-described by the hard core van der Waals model [10],

$$p_B(t) = \left[p(t) + \frac{2S}{R(t)}\right]\left[\frac{R_\infty^3 - h^3}{R(t)^3 - h^3}\right]^\gamma \tag{2}$$

and the external pressure, $p(t)$, is given by

$$p(t) = p_\infty + p_{s0}f(t) \qquad (0 \leq f(t) \leq 1) \tag{3}$$

in which R_∞ is the bubble radius for which the pressure inside the bubble becomes equal to the pressure of water at the position sufficiently far from the bubble (i.e., external pressure), p_∞, h is the actual radius by taking into account for the liquid molecules covolume [10], and γ is the specific heat of the liquid. $p_{s0}f(t)$ is the time-varying pressure wave originating from the formation of NBs at the surrounding NPs with p_{s0} and $f(t)$ being the peak pressure and the normalized function of the pressure wave, respectively. If the contribution of the pressure wave emitted by the neighboring NPs is negligible, then, $p_{s0}f(t) \to 0$ and hence $p(t) \to p_\infty$, which is the well-known situation of an isolated bubble.

For our specific cases presented in Fig. 5, we solve eqs 1-3 for $S = 7.2 \times 10^{-2}$ J/m^2, $\rho_L = 1000$ kg/m^3, $h = R_\infty/9.174$, $\gamma = 1.13$ (corresponding to the adiabatic evolution of the bubble), $p_\infty = 0.1$ MPa, and $R(t = -\infty) = 0.05$ μm for the initial size of our Ag NPs with the peak pressure p_{s0} and the initial bubble speed $\dot{R}$ being the parameters to be fitted to the experimental results. The temporal form of $f(t)$ is assumed to be a sine-squared function with the rise time of 3 ns until it reaches its peak pressure value, and then kept to be constant for the duration of $\sim$10 ns (Fig. 5a) which is the time scale of our interest. For the NP solution with different number densities the value of p_{s0} is consistently scaled, based on the fact that the peak value of the pressure wave is inversely proportional to the square of the inter-particle distance.

Supplementary Note 2

Procedure to ensemble-average the numerical results

By numerically solving the appropriately modified RP equation (Supplementary Note 1) we obtain the time-dependent change of the NB radius under the pressure wave. In our case, however, there is one more complication we must take care of: Since the employed laser pulse has a nanosecond duration, different NBs may be born from different NPs at different times during the laser pulse, and as a consequence, what we experimentally observe is an ensemble average of the extinction cross sections associated with those NBs. After these considerations, we perform the bubble calculations at a certain number density of NPs under the influence of the pressure wave as assumed in Fig. 5a for different onset times of the NBs, and cast the calculated bubble radius into the extinction cross section. The extinction cross sections are then ensemble averaged with weighting factors proportional to the number of NBs born at that moment, which is assumed to be proportional to the instantaneous laser intensity. The ensemble-averaged extinction cross section is finally recast into the bubble radius. Note that this kind of ensemble effect, which tends to blur the pressure wave effects, does not exist if the employed laser pulse has a much shorter (picosecond or femtosecond) duration [9] or a single NB is produced by laser ablation onto a solid target placed in liquid [6–8].

Supplementary References

[1] Brennen, C.E. Cavitation and Bubble Dynamics ; Oxford University Press: Oxford, 1995.

[2] Plesset, M.S. The dynamics of cavitation bubbles. *J. Appl. Mech.* **16**, 277-282 (1949).

[3] Petkovšek, R., Gregorčič, P. A laser probe measurement of cavitation bubble dynamics improved by shock wave detection and compared to shadow photography. *J. Appl. Phys.* **102**, 044909 (2007).

[4] Vogel, A., Busch, S. Shock wave emission and cavitation bubble generation by picosecond and nanosecond optical breakdown in water. *J. Acoust. Soc. Am.* **100**, 148-165 (1996).

[5] Vogel, A., Linz, N., Freidank, S., Paltauf, G. Femtosecond-laser-induced nanocavitation in water: implications for optical breakdown threshold and cell surgery. *Phys. Rev. Lett.* **100**, 038102 (2008).

[6] Evans, R., Camacho-López, S., Pérez-Gutiérrez, F. G., Aguilar, G. Pump-probe imaging of nanosecond laser-induced bubbles in agar gel. *Opt. Express* **16**, 7481-7492 (2008).

[7] Sasaki, K., Nakano, T., Soliman, W., Takada, N. Effect of pressurization on the dynamics of a cavitation bubble induced by liquid-phase laser ablation. *Appl. Phys. Express* **2**, 046501 (2009).

[8] Thornton, B., Sakka, T., Takahashi, T., Tamura, A., Masamura, R., Matsumoto, A. Spectroscopic measurements of solids immersed in water at high pressure using a long-duration nanosecond laser pulse. *Appl. Phys. Express* **6**, 082401 (2013).

[9] Kotaidis, V., Plech, A. Cavitation dynamics on the nanoscale, *Appl. Phys. Lett.* **87**, 213102 (2005).

[10] De Giacomo, A., Dell'Aglio, M., Santagata, A., Gaudiuso, R., De Pascale, O., Wagener, P., Messina, G. C., Compagnini, G., Barcikowski, S. Cavitation dynamics of laser ablation of bulk and wire-shaped metals in water during nanoparticles production. *Phys. Chem. Chem. Phys.* **15**, 3083-3092 (2013).
